# The Impact of Winter and Spring Temperatures on Temperate Tree Budburst Dates: Results from an Experimental Climate Manipulation

**DOI:** 10.1371/journal.pone.0047324

**Published:** 2012-10-10

**Authors:** Yongshuo H. Fu, Matteo Campioli, Gaby Deckmyn, Ivan A. Janssens

**Affiliations:** Department of Biology, University of Antwerp, Wilrijk, Belgium; Odum School of Ecology, University of Georgia, United States of America

## Abstract

Budburst phenology is a key driver of ecosystem structure and functioning, and it is sensitive to global change. Both cold winter temperatures (chilling) and spring warming (forcing) are important for budburst. Future climate warming is expected to have a contrasting effect on chilling and forcing, and subsequently to have a non-linear effect on budburst timing. To clarify the different effects of warming during chilling and forcing phases of budburst phenology in deciduous trees, (i) we conducted a temperature manipulation experiment, with separate winter and spring warming treatments on well irrigated and fertilized saplings of beech, birch and oak, and (ii) we analyzed the observations with five temperature-based budburst models (Thermal Time model, Parallel model, Sequential model, Alternating model, and Unified model). The results show that both winter warming and spring warming significantly advanced budburst date, with the combination of winter plus spring warming accelerating budburst most. As expected, all three species were more sensitive to spring warming than to winter warming. Although the different chilling requirement, the warming sensitivity was not significantly different among the studied species. Model evaluation showed that both one- and two- phase models (without and with chilling, respectively) are able to accurately predict budburst. For beech, the Sequential model reproduced budburst dates best. For oak and birch, both Sequential model and the Thermal Time model yielded good fit with the data but the latter was slightly better in case of high parameter uncertainty. However, for late-flushing species, the Sequential model is likely be the most appropriate to predict budburst data in a future warmer climate.

## Introduction

Leaf phenology is a key driver of canopy development, tree growth, ecosystem carbon and water balance, and species distribution [1], [2], [3], [4], [5]. In the Northern hemisphere, a clear advancement of spring tree phenology, paralleling the recent increase in surface temperature, has been well documented [6], [7], [8], [9], [10], [11], [12], [13]. However, this observed correlation between temperature and leaf flushing date cannot be extrapolated to simulate the future phenology changes in a warmer world, because the mechanisms underlying the budburst process are far from fully understood [14], [15].

Winter temperatures (referred from here on as chilling) that determine the release from dormancy in winter [16] and spring warming temperatures (referred to as forcing) that accelerate bud development following the release from dormancy are both acknowledged to influence spring phenology [16], [17], [18], [19]. However, climate warming is expected to have a contrasting effect on chilling and forcing. Warmer springs logically accelerate the accumulation of forcing and hence advance the timing of budburst. Warmer winters may also advance budburst timing if they contribute to forcing, but warmer winters may also reduce the accumulated chilling. This might have no effect on budburst (if the chilling requirement is already fulfilled early in winter), but could also delay it (if the tree chilling requirement remains unfulfilled in warmer conditions) [14], [15], [20], [21], [22]. Thus, future climate warming might not result in a straightforward advancement of budburst because of contrasting effects of warming on the accumulation of chilling and forcing, and the extent of warming in winter and spring is likely to differ [23]. To accurately simulate the budburst process in a changing climate, more information on the impact of warming on chilling and forcing phases, separately, as well as their combined effect on tree phenology, is needed.

**Table 1:** The symbols and units of the variables and parameters used in the equations of the studied models.

Model Symbole	TTM	SM	PM	AM	UM	Description	Units
Variables							
CU						Chilling unit	-
FU						Forcing unit	-
S_c_						State of chilling	CU
S_f_						State of forcing	FU
R_c_						Rate of chilling	CU
R_f_						Rate of forcing	FU
T						Mean daily air temperature	°C
D						Day of the year	day
BB						Day of budburst	day
F^*^	(a)	(a)	(a)		(a)	Forcing threshold	FU
Parameters							
				t_1c_		Start day of chilling period	day
				t_2c_	t_2c_	End day of chilling period	day
	t_1f_			t_1f_		Start day of forcing period	day
	T_b_			T_b_		Critical temperature to induce forcing start	°C
				T_c_		Critical temperature to accumulate chilling units	°C
		C^*^	C^*^		C^*^	Chilling threshold	CU
		T_min_	T_min_			Minimum temperature for rate of chilling	°C
		T_opt_	T_opt_			Optimal temperature for rate of chilling	°C
		T_max_	T_max_			Maximum temperature for rate of chilling	°C
		a, b, c	a, b, c, K_m_	a, b, c	C_a,_ C_b,_ C_c,_ w, k, F_b_, F_c_,	Constant	-

(a) Parameter for the model.

Over the years, a wide variety of budburst models have been developed. The simple one phase models that take only the forcing phase into account, are generally accepted to predict the budburst timing well [24], [25]. Nevertheless, very good performance of two-phase models (including both chilling and forcing phases) was recently reported, especially for species flushing later in spring [13], [22], [26]. However, most of these studies used historical phenology observations, and were seldom based on actual climate manipulation experiments in which climate can be forced beyond the currently occurring climatic envelope. Therefore, phenology models may generate high uncertainty when predicting future phenology shifts in response to global warming. To study the budburst response to the anticipated climate warming and to estimate the accuracy of the currently used temperature-based budburst models, a manipulative experiment was designed using climate-controlled greenhouses. In particular, the effect of warming on the chilling and forcing phases was assessed by considering two different warming periods in a factorial design. Three tree species were selected: birch (*Betula pendula* L., with budburst around mid April in Belgium), oak (*Quercus robur* L., with budburst in early May) and beech (*Fagus sylvatica* L., with budburst in mid May [27]). These species were chosen to reflect a different pattern of temperature requirement during the budburst process, particularly between the pioneer birch and the late successional beech. For example, Murray *et al* [15] reported larger chilling requirement for late flushing species (like beech, 150 chilling days) than early flushing species (like *Crataegus monogyna*, 80–100 chilling days). The Belgian phenology network dataset [28] indicates chilling requirement of 120 chilling days for beech, 100 chilling das for oak and 50 chilling days for birch (Fu unpublished). We expected the warming-induced budburst advancement to be more pronounced for birch than for beech, as the larger chilling requirement of the latter might be partially unfulfilled in warmer winter conditions. Oak is expected to have an intermediate pattern. Five temperature-driven budburst models were chosen to reproduce budburst timing: the Thermal Time model (*TTM*) [19], the Parallel model (*PM*) [29], [30], [31], the Sequential model (*SM*) [31], [32], [33], the Alternating model (*AM*) [15], [19], [31], and the Unified model (*UM*) [34]. We sought to answer the following questions: (i) Does budburst respond differently to winter warming than to spring warming? and (ii) which temperature-driven model is best at reproducing budburst dates in manipulated warming conditions?

**Figure 1:**
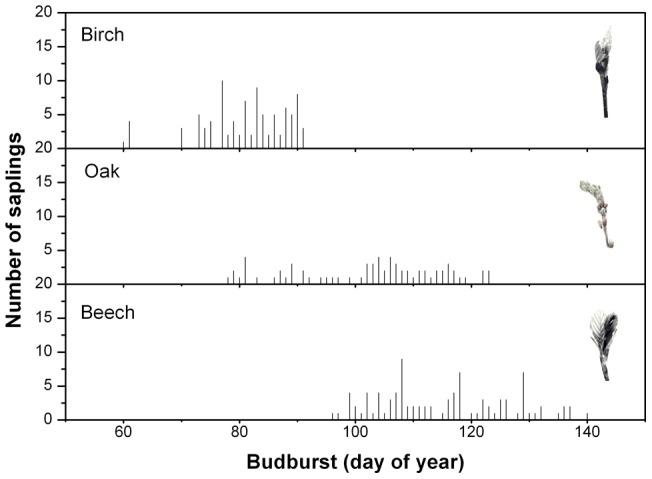
Frequency distribution of budburst dates of the experimental trees for the three studied species. Frequency distribution of budburst dates of the experimental trees for the three studied species. The number of saplings for a given budburst date is presented on the Y-axis. The small photos on the upper right part of each panel visually show the stage of leaf development when we considered budburst to have occurred.

## Materials and Methods

### Warming experiment

The experiment was conducted at the Drie Eiken campus of the University of Antwerp (Belgium, 51^°^19"N, 4^°^21"E). The climate in Belgium is characterized by mild winters and cool summers, with a long-term average annual air temperature of 9.6°C, and mean monthly air temperatures between 2.2°C (January) and 17.0°C (July). Annual precipitation averages 776 mm, equally distributed throughout the year.

**Figure 2:**
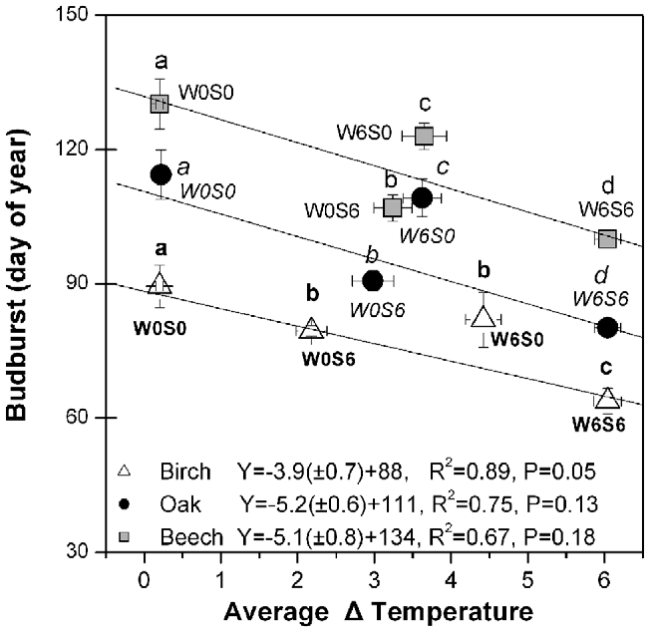
Budburst response to different warming treatments. Budburst response to different warming treatments: W0S0: no warming; W6S6: winter and spring warming; W6S0: winter warming only, and W0S6: spring warming only (winter is from December 1 2009 to February 22 2010 and spring from February 22 to budburst date). The delta temperature was calculated as the average difference between treatments and outside controls from December 1^st^ to the day of observed budburst for three warming treatments. Because the period with warming in spring was shorter than the period with warming in winter, the W0S6 treatment experienced less warming on average than the W6S0 treatment. Especially for birch, the earliest flushing species, this difference was pronounced. The different letter close to the symbols denote a significant difference (at P<0.05) among treatments for each species, separately.

One-meter-high saplings of *Betula pendula* L., *Quercus robur* L. and *Fagus sylvatica* L. of a local provenance were used in this experiment. Up to the start of our manipulation experiment, all nursery-grown saplings were subjected to uniform conditions, i.e. equal fertilization, irrigation and light conditions. Saplings were transplanted to plastic pots (diameter 25 cm, depth 30 cm) in December of 2009. The pots were filled with a loamy sand top soil with a pH of 5.5 and 1.9% soil organic carbon (determined by the Belgian National Soil Service, Belgium). The soil organic carbon was determined by the dichromate method (ISO 14235), and the pH of the soil was measured using standard methods (ISO 10390). Before moving into the greenhouses, the saplings were fertilized again. The composition of the slow release fertilizer was 13-10–20 for nitrogen, phosphate and potassium (all in %). The recommended fertilizer dose was 100 g m^−^^2^; therefore 20 g of fertilizer was added to each sapling (pot area  = 0.2 m^2^). For this experiment we used 8 climate-controlled experimental chambers. The interior surface area was 150×150 cm, the height at the north side 150 cm and at the south side 120 cm. Each chamber has a roof fitting the upper opening. The four sides were made of polyethylene film (200 mm thick), whereas the roof of polycarbonate plate (4 mm thick). All sides and roof were sunlit, colorless and UV transparent. The chambers can be artificially warmed in a controlled manner up to 9°C, using a centralized heating system of continuous (day and night) warming above fluctuating ambient air temperature [35]. Four chambers were maintained at ambient temperature for the entire duration of the experiment (from December 1^st^ 2009 till budburst in spring 2010), whereas another four chambers were continuously warmed by 6°C. Six saplings of beech, oak and birch were placed in each chamber (totaling 48 saplings per species). Half of the saplings were left in the same chamber for the duration of the experiment. Saplings that remained in the control chambers constituted the W0S0 treatment, whereas saplings that remained in the warmed chambers constituted the W6S6 treatment. The other three saplings per greenhouse were moved from the control chambers to the warmed chambers (W0S6) or vice versa (W6S0) when the ambient mean daily temperature was above 5°C for five-days continuously i.e. on February 22^nd^ in 2010. A forcing temperature threshold of 5°C is commonly used in temperate regions [11], [15], [31]. The moving of these saplings provide the distinct warming treatments, i.e. only winter warming (W6S0) and only spring warming (W0S6). Overall, we considered four treatments (W0S0, W6S0, W0S6, W6S6), with level of replication of (*n*) of four, i.e. four chambers per treatment. Each replicate value is the average of three within-chamber observations (three saplings of each species per chamber). In total, we had 12 saplings per treatment. The saplings were watered once or twice per week. The saplings were watered as soon as the topsoil appeared dry. Because the warmed chamber resulted in faster evapotranspiration, these saplings were irrigated more frequently than that in control. In this way, the soil retained sufficient water, and would not confound the temperature effect on the budburst process, as Morin *et al* [21] reported that the soil water content did not affect the leaf unfolding phenology of oak seedlings. Eight temperature sensors (Siemens, type QFA66, Germany) were used to continuously monitor (logging time 30 minutes) the inside air temperature of each chamber, and also the outside reference temperature was monitored. The chambers provided a stable warming treatment (regressions of chamber temperature vs. field temperature were highly significant (P<0.001) and with average R^2^ above 0.95) and actual warming was within ±5% of the prescribed value [35].

**Table 2:** Root mean square error (RMSE), model efficiency (ME) and Akaike's Information Criterion (AIC) of model validation for the five models and the three study species.

Models	Internal dataset						External dataset						whole dataset		
	beech		oak		birch		beech		oak		birch		beech	oak	birch
	RMSE	ME	RMSE	ME	RMSE	ME	RMSE	ME	RMSE	ME	RMSE	ME	AIC		
TTM	4.1	0.75	3.1	0.94	3.0	0.82	5.6	0.59	**3.7**	0.88	3.7	0.77	75.0	**57.5**	**57.5**
SM	**3.4**	**0.82**	**2.9**	**0.95**	**2.9**	**0.84**	**4.0**	**0.79**	**3.7**	**0.91**	**3.4**	**0.80**	**72.7**	67.2	65.0
PM	6.3	0.41	4.7	0.85	4.4	0.62	6.5	0.47	5.0	0.85	4.9	0.66	98.6	87.5	81.5
AM	6.5	0.41	4.0	0.9	5.3	0.48	6.5	0.46	4.7	0.85	6.2	0.33	88.1	72.2	85.6
UM	4.2	0.74	5.8	0.79	4.5	0.61	4.8	0.7	5.8	0.79	5.5	0.56	81.2	91.1	86.4

‘Internal dataset’ indicates that calibration and validation were done on the same dataset, whereas ‘external dataset’ indicate that calibration and validation were done on independent datasets. The AIC were calculated on the whole dataset. TTM is Thermal Time model, SM is sequential model, PM is Parallel model, AM is Alternating model and UM is Unified model. The smallest RMSE and highest ME values for each species are in bold.

### Phenology measurement

Phenological observations were made on the terminal bud of each sapling, according to the following phenology scale: (1) undeveloped bud: bud still in winter dormancy; (2) swollen bud: green or elongated bud or bud with broken scales, i.e. with the leaf tip becoming visible but still forming a single bud tip; (3) bud break: leaf bases still hidden in bud scales but leaf tips detached from the bud axis, and (4) leaf unfolded: the entire leaf blade and the leaf stalk are visible. Monitoring started on February 1 and was repeated every 3 days (within stages 1 and 2) and every 2 days (within stages 3 and 4) always at the same time (2:00–3:00 PM). In this study, we used the starting date of stage 3 to determine budburst date as shown in Figure 1.

**Figure 3:**
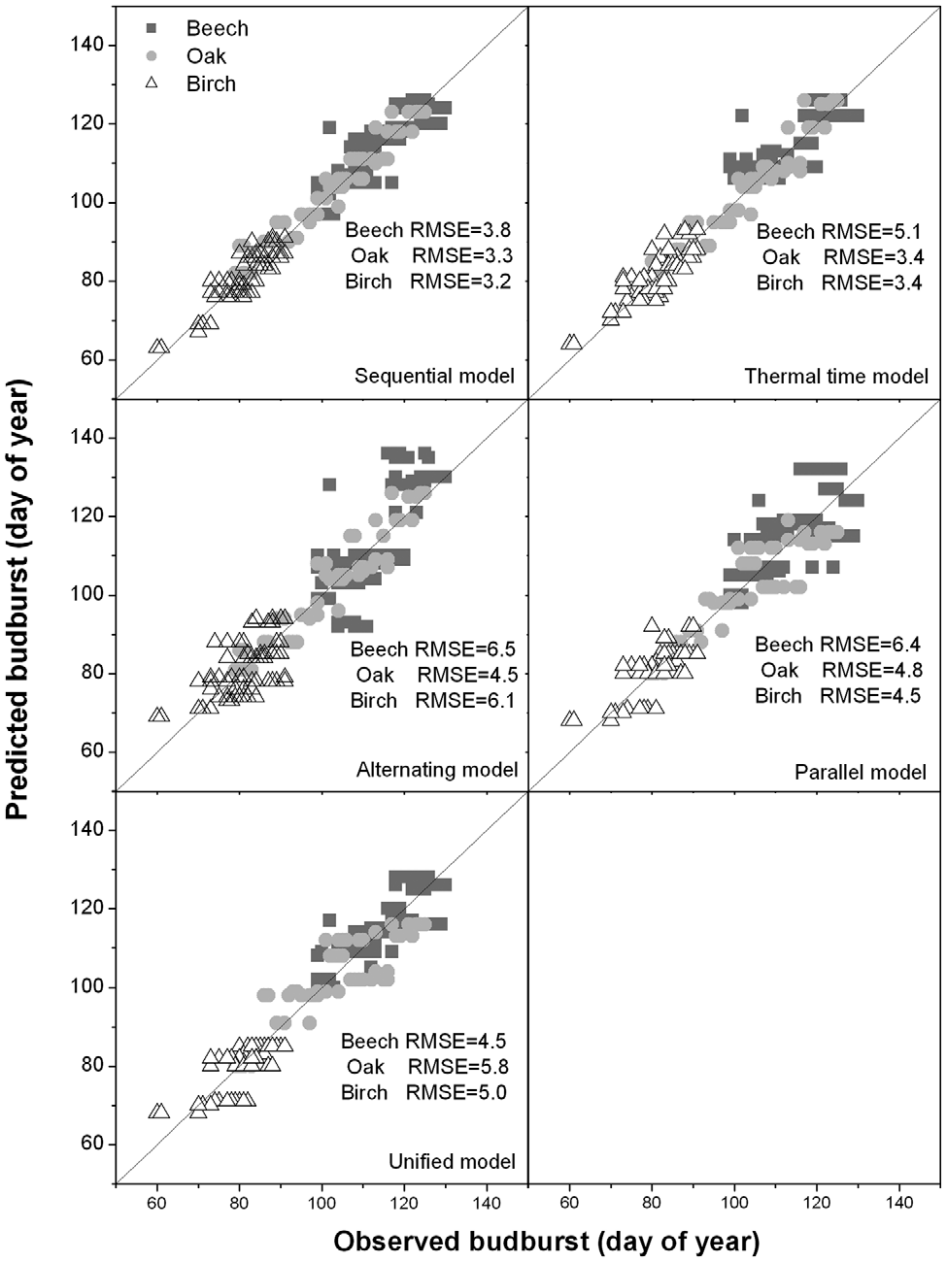
Comparison of the observed budburst dates with the predicted values for five models. Comparison of the observed budburst dates with the predicted values for five models fitted on the whole observation dataset. Data are for three species. The diagonal line is the 1∶1 line, whereas RMSE is the Root Mean Square Error.

### Models used

Five models were parameterized to predict the budburst date. In the models, the effect of temperature is accounted by calculating (daily) the rate of forcing (*R_f_*) and rate of chilling (*R_c_*), both as functions of the daily air temperature (*T*). These functions differ among models. *R_f_* and *R_c_* determine the state of forcing (*S_f_*) and the state of chilling (*S_c_*), respectively:

**Figure 4:**
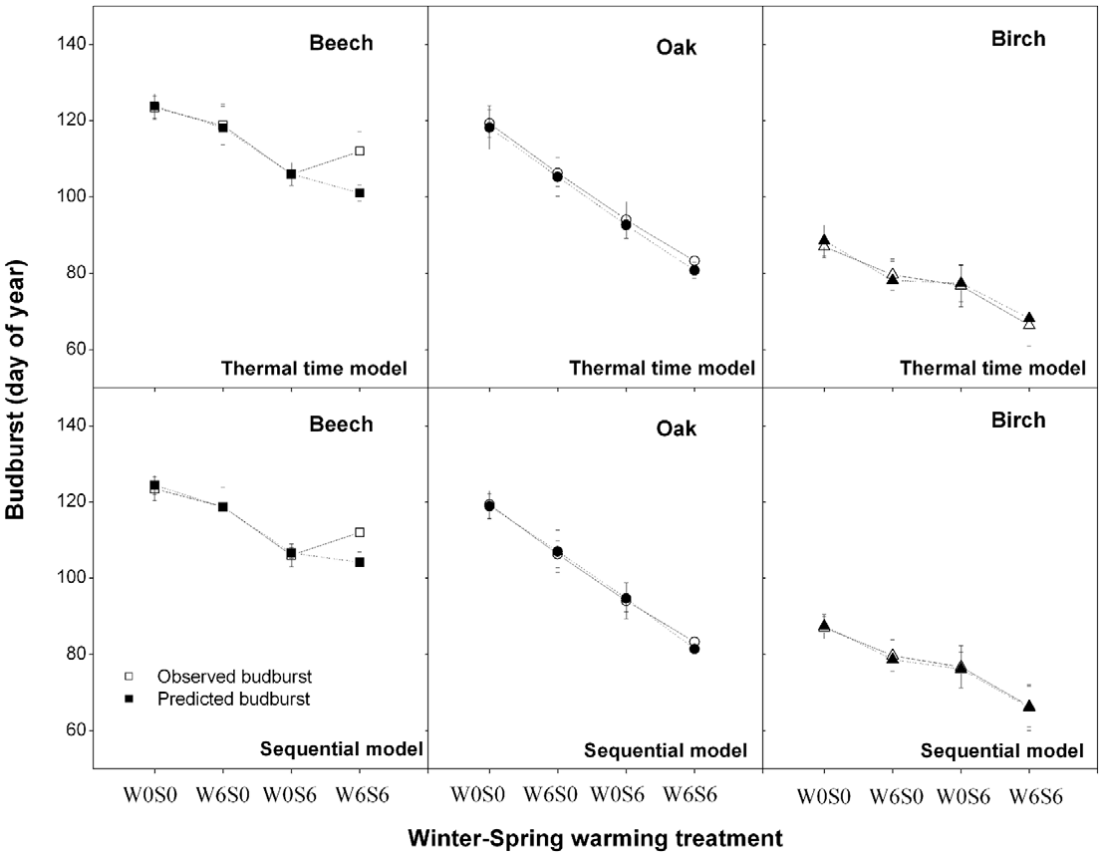
Modeled against observed budburst dates for different warming treatments. Modeled against observed budburst dates for different warming treatments (W0S0: no warming; W6S6: winter and spring warming; W6S0: winter warming only, and W0S6: spring warming only; winter is from December 1 2009 to February 22 2010 and spring from February 22 to budburst date) when using the Sequential model and the Thermal time model for each species. The models were fitted on the whole observation dataset. The observed budburst dates are represented by open symbols and the predicted budburst dates are represented by solid symbols.



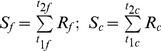
(1)where *t_1f_* and *t_1c_* is the initial day of the forcing- and chilling period, respectively, and, similarly, *t_2f_* and *t_2c_* is the final day of the forcing- and chilling period, respectively. The budburst day (*BB*) occurs when *S_f_* reaches a forcing threshold F^*^, whereas, in the models that account for the chilling temperature, the start of forcing is triggered when *S_c_* reaches the chilling threshold C^*^.



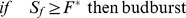
(2)

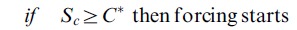
(3)where *D* is the day of the year. For the model names and model parameters, the terminology follows original works (cited below). For each model, a complete list of model parameters and variable used is reported in table 1.

***Thermal time model (TTM)*** [19] In the *TTM*, the forcing period starts on a fixed day (*t_1f_*), and *R_f_* is computed when the air temperature is above a critical temperature (*T_b_*) by using a linear relationship as follows (Eqs.4).
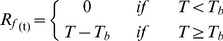
(4)


The *TTM* ignores any eventual role of chilling. TTM has 3 parameters (*t_1f_*, *T_b_*_,_ F^*^_)._

***Sequential model (SM)*** [30], [31], [32]. The *SM* model uses a triangular chilling function (Eq 5) and a sigmoid forcing function (Eq 6):
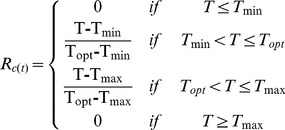
(5)

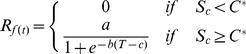
(6)where *T_min_, T_max_, T_opt_* are the minimal, maximal and optimal temperature for chilling period, and *a, b, c* are fitting parameters. The *SM* starts accumulating forcing units only when a sufficient amount of chilling has occurred (Eq. 3). *SM* has eight parameters (T_min_, T_max_, T_opt,_ a, b, c, C^*^ and F^*^).

***Parallel model (PM)*** [29], [30], [31] The *PM* assumes that the forcing phase can take place even during the chilling phase. *R_c_* is calculated as in *SM* (Eq. 6), whereas *R_f_* is calculated with a modification of Eq. 5 of *SM*:
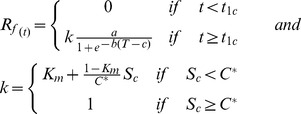
(7)Where *K_m_* is a model parameter and C^*^ is the threshold indicating complete chilling. *PM* has one more parameter (*K_m_*) than *SM*, thus nine in total.

***Alternating model (AM)*** [15] The *AM* has the same rate of forcing as the *TTM* (Eq. 4) but a fixed forcing start on January 1^st^ [15]. The chilling rate equals the number of chilling days, i.e. days with average temperature less than a chilling threshold *T_c_* (Eq. 8), with start of chilling fixed on November 1^st^ [15].
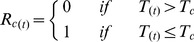
(8)


The major difference between *AM* and the other models is the definition of F^*^ (Eq. 2), which in *AM* is not a constant parameter but a negative exponential function of the state of chilling (Eq.9) [19], [30]. In this way, flexibility is introduced in modeling the budburst process as the forcing period is controlled by the chilling period.
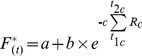
(9)Where a, b and c are fitting parameters. AM has eight parameters (*t_1f_* , *t_1c_*, *t_2c_*, T_b,_ T_c_, a, b, c).

#### Unified model (UM [34])

The unified model combines features of the other models and merges the equations for *R_c_* and *R_f_* into one sigmoid equation:
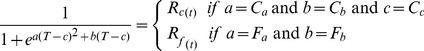
(10)where *C_a_, C_b_* and *C*_c_ are chilling rate parameters, *F_b_* and *F_c_* are forcing rate parameters. In *UM*, *t_1c_* is set on September 1^st^ [34]. The forcing units start to accumulate when a sufficient amount of chilling has occurred (C^*^), and F* is calculated with an exponential function of *R_c_* similarly to Eq. 14 from *t_1c_* to *t_2c_*,
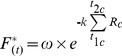
(11)where k, w and t2c are model parameters. The Unified model has nine parameters (Ca, Cb, Cc, Fb, Fc ,C*, k, w, t2c).

### Model calibration and validation

The models were parameterized by Bayesian method using the version of Markov Chain Monte Carlo known as the Metropolis-Hastings random walk [36]. This method has already been used for the parameterization of the same phenology models investigated here [37]and a detailed methodological description can be found in [28]. Because of lack of information on the parameters, flat distributions for parameters were defined as the prior parameter distribution [38]. Datasets used for parameterization and validation comprised all the saplings used in the winter-spring warming experiment described above (48 saplings per species) plus additionally 36 sapling per species. The latter saplings were studied in a parallel experiment that used chambers warmed by 2°C in winter in the same experimental platform described above and moved after February 22^nd^ to control conditions (W2S0), +2°C (W2S2) and +6°C (W2S6) in another experimental platform [39]. As a result, a wide range of budburst dates were available for each species (Figure 1), which benefit the budburst modeling work. The models were parameterized and validated against the whole dataset (internal validation) and/or parameterized on a randomly selected half of the dataset and tested against the other half (external validation). The model performances were evaluated by the Root Mean Square Error (*RMSE*), Model Efficiency (*ME*) and Akaike's Information Criterion (*AIC*):



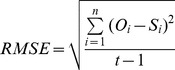
(12)




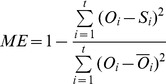
(13)




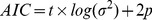
(14)Where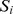 is the model prediction, 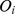 is the experimental data, and *t* is the number of observations, *p* is the number of model parameters plus one and 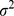 is the residual sum of squares divided by *t*.

### Statistics and calculations

Pearson's correlation coefficient (r) was calculated to determine the correlation between the observed and predicted budburst dates. One way ANOVAs were used to evaluate the significance level of the budburst differences among treatments. All statistical analyses were conducted using SPSS 16.0 (SPSS Inc., Chicago, IL, USA).

## Results

### Budburst dates under warming

A wide range of budburst dates was obtained for each of the three studied species (Figure 1). Independently of the treatment, birch flushed earlier than oak, and oak, on average, exhibited earlier budburst than beech (Figure 1). Compared with the budburst date range of birch (day of year, DOY, 60–95), the budburst date range was wider in oak (47 days: DOY 78–125) and beech (45 days: DOY 95–140) (Figure 1). The continuously warmed W6S6 treatment resulted in the earliest budburst dates, while in the control chambers (W0S0) the latest budburst dates were observed (Figure 2).

### Budburst response to winter and spring warming

Warming advanced the timing of budburst in all three study species (Figure 2). However, the observed temperature sensitivity of budburst, estimated here as the number of days advancement in budburst over the average increase in temperature over the period December 1^st^ to bud burst, differed substantially among species (slopes in Figure 2). Oak and beech exhibited the highest sensitivity to warming (>5 days earlier per °C warming), and birch the lowest (3.5 days °C^−1^), but these species differences in sensitivity were not statistically significant (P = 0.41 for birch and oak; P = 0.53 for birch and beech; P = 0.79 for oak and beech).

Although all warming treatments elicited earlier budburst relative to the control population, and this in all three species, substantial differences in temperature sensitivity were found between trees that were warmed in winter versus spring (Figure 2). In all three species, budburst in the W6S0 treatment (warmed only in winter), occurred later than in the W0S6 treatments (warmed only in spring), despite the former having been warmed for a longer period. Figure 2 clearly shows that all W6S0 treatments are above the average temperature sensitivity line, while W0S6 treatments are well below the line, confirming that budburst in all three species is much more sensitive to spring warming that to winter warming.

### Model comparison

All models yielded low values of *RMSE* (less than seven days) when fitted on the whole observation dataset (Figure 3). The best-fit parameters values of each model (those with the maximum likelihood as determined with Bayesian calibration) are provided in appendix table S1. For all models, cross-validation showed low values of *RMSE* and high values of *ME* in both internal and external validations across the studied models (Table 2). This indicates that all models can be used to reproduce the budburst date, although caution should be taken when applying *PM* to beech and *AM* to beech and birch (which showed *RMSE*  = 5–7 days and *ME*  = 0.3–0.5) (Table 2). The Sequential model (*SM*) performed best across the three studied species (lowest *RMSE* and highest of all models; Table 2). However, the Sequential model has more parameters and therefore the *AIC* supports the *TTM* as best model for oak and birch (*AIC* = 57.5 for both species), while *SM* is still selected for beech (*AIC* = 72.7).

As the *SM* and the *TTM* were the best models, the performances of these two models were checked for the different warming treatments, separately (Figure 4). The warming treatments resulted in small differences in model performance. Although the *SM* performed slightly better, both *SM* and *TTM* successfully reproduced the budburst date of birch and oak across all treatments. For beech, both *TTM* and *SM* successfully predicted budburst data in W0S0, W0S6 and W6S0, but yielded earlier budburst than observed in W6S6. This difference was small for *SM* (3 days), but relevant for *TTM* (10 days).

## Discussion

### Budburst response to warming

The experimentally manipulated separate warming in winter and spring demonstrated that both winter and spring warming significantly hasten budburst. The strong effect of spring warming on budburst advancement was expected. On the other hand, for all study species, we did not observe a delayed budburst response (rather advancement) to winter warming compared to the control treatment, but did observed later budburst dates in winter warming treatment than in spring warming. We propose two hypotheses to explain the positive effect of winter warming on budburst. First, the chilling requirement of the investigated species was met even under warming conditions. In fact, chilling occurred even in the warmed treatments (data not shown), and some additional chilling might have occurred between bud set [40] and the start of the warming experiment on December 1^st^. Second, the delaying effect due to unfulfilled chilling was offset and outweighed by a positive effect of winter warming. The positive effect of winter warming (assumed in both hypotheses) might be indirect and related to an earlier start of the forcing phase in warmed saplings (thus before February 22^nd^).

Our expectations on budburst advancement to be more pronounced for birch than for beech and oak were not confirmed as the budburst temperature sensitivities were non-significantly different among the three study species. In addition, the fact that the advancing effect of strong winter warming, i.e. the W6S6 treatment, on budburst is less for beech, indicate that photoperiod might also play a role in determining the switching between chilling and forcing in late successional species, whose phenology is well known to be affected by photoperiod [41], [42], [43], [44], [45]. The investigated saplings were well fertilized and irrigated throughout the experiment, in order to eliminate a confounding influence of soil water and nutrient availabilities. The future warming may likely lead to changes of precipitation [46], thus altering soil water conditions. Therefore, spring growth conditions may be affected by soil moisture as well as warmer temperatures in future warming conditions.

### Predicting future phenology shifts

The model comparison results suggest that temperature-based models can successfully reproduce the date of budburst, and confirmed that temperature is still the main driver of the budburst process under future warming climate. In this study, the one-phase Thermal Time model (without chilling requirement) performed as good as the Sequential model and better than other two-phase models, suggesting that the chilling phase does not play a decisive role in the budburst process of these species under the investigated environmental conditions in this study. The poor performance of the *PM, AM* and *UM* model may be link to the fact that these models have many parameters, introducing a risk of over-parameterization. This result is consistent with other model comparison studies using historical phenology datasets [24], [25], [37]. This finding further suggests that budburst is mainly controlled by the forcing phase (i.e. budburst can be modeled with warmth units accumulated since a fixed starting date), and negative effects in response to the reduced chilling are only marginally important. However, caution should be raised. First, the chilling requirement for *Betula pendula* L. and also for *Quercus robur* L. is probably much smaller than the chilling requirement of *Fagus sylvatica* L.[15]. Therefore sufficient chilling may have occurred between bud set and the start of the temperature manipulation [40]. Under these conditions, the similar performances for the *TTM* and *SM* are not surprising. On the other hand, the fact that *SM* performed markedly better than *TTM* for W6S6 in beech (and to a lesser extent in oak too), indicates that chilling did influence budburst in the late-flushing successional species. A better performance of two-phase models for late-flushing / late-successional species was also pointed out in other studies [13], [26].

In this study, we found that both the *SM* and *TTM* were unable to accurately predict the budburst date in the W6S6 treatment, although the *SM* model approximated the observations relatively well. A hypothesis to explain this result may be a possible effect of photoperiod in constraining the advancement of spring phenology, as both *SM* and *TTM* predict earlier budburst than observed, but only in the warmest treatments that exhibited budburst earliest. Thus, the determining factor in budburst advancement may have shifted from temperature requirements to a light signal under extreme warming conditions (e.g. +6°C) [30], [43]. The short photoperiod in late winter and early spring may prevent budburst to protect the trees from frost damage. The important role of photoperiod (especially for late-flushing and late-successional species such as beech) and light quality (red to far-red light) in the budburst process is well established [30], [47]. and photoperiod has indeed been shown to affect the phenology of some species, especially for late-flushing and late-successional species such as beech [41], [48].

Previous comparisons of the different models suggested that no model is superior for all species and should be put forward as a consensus model [22], [28], [49], [50]. In this study, we found that *SM* is the best model for beech, and both *SM* and *TTM* were very good models for oak and birch, but with the *TTM* slightly better for the latter two species in case parameter uncertainty is high. The *SM* model therefore might be the most appropriate model to predict budburst data in a future warmer climate, especially for late-flushing species.

Parameterization of phenology models is troublesome. Fitting many parameters with relatively few data is an inherently difficult process and, although model predictions might be correct, some model parameter values might be not fully realistic. Furthermore, complex phenology models might be over-parameterized. For instance, some parameters of the Unified model can be correlated or not relevant (corresponding to low model sensitivity) [28]. This lowers the quality of the parameterization procedure and maybe explains why simpler models were found to have a better fit than the parameter-rich models. Improvements on model parameterization might comprise treating correlated parameters as clusters, model insensitive parameters as constants and, in particular, determining experimentally the model parameters with biological meaning.

## Supporting Information

Table S1 **The best-fit parameters values of each model.** The best-fit parameters values of each model, those with the maximum likelihood as determined with Bayesian calibration.
(DOCX)
