# Supplementary material for: The Impact of Winter and Spring Temperatures on Temperate Tree Budburst Dates: Results from an Experimental Climate Manipulation
Source: PLoS One. 2012 Oct 10;7(10):e47324. doi: 10.1371/journal.pone.0047324 (PMC3468505; doi:10.1371/journal.pone.0047324)
Supplement: Table S1 — The best-fit parameters values of each model. The best-fit parameters values of each model, those with the maximum likelihood as determined with Bayesian calibration. (DOCX) [file pone.0047324.s001.docx]

Table S1. The best-fit parameters values of each model, those with the maximum likelihood as determined with Bayesian calibration.

| models | t_1c_ | T_c_ | T_max_ | T_min_ | T_opt_ | C^*^ | t_2c_ | t_1f_ | T_b_ | a | b | c | F^*^ | k_m_ | k | w |
| --- | --- | --- | --- | --- | --- | --- | --- | --- | --- | --- | --- | --- | --- | --- | --- | --- |
| ***TTM*** |  |  |  |  |  |  |  |  |  |  |  |  |  |  |  |  |
| beech |  |  |  |  |  |  |  | 1March | 2.5 |  |  |  | 607.9 |  |  |  |
| oak |  |  |  |  |  |  |  | 2Feb. | 5.4 |  |  |  | 549.5 |  |  |  |
| birch |  |  |  |  |  |  |  | 8Feb | 5.2 |  |  |  | 264.3 |  |  |  |
| ***SM*** |  |  |  |  |  |  |  |  |  |  |  |  |  |  |  |  |
| beech | 1Nov. |  | -4.6 | 8.0 | 14.4 | 447.1 |  |  |  | 29.7 | 0.09 | 10.56 | 1586.6 |  |  |  |
| oak | 1Nov. |  | -59.5 | 10.9 | 17.8 | 398.4 |  |  |  | 47.7 | 0.15 | 14.72 | 1130.6 |  |  |  |
| birch | 1Nov. |  | -10.8 | 8.6 | 22.9 | 403.6 |  |  |  | 66.1 | 0.35 | 7.88 | 848.2 |  |  |  |
| ***PM*** |  |  |  |  |  |  |  |  |  |  |  |  |  |  |  |  |
| beech |  |  | -9.69 | -1.69 | 17.86 | 430.49 |  |  |  | 87.26 | -0.4 | -9.63 | 31.36 | 0.06 |  |  |
| oak |  |  | -3.87 | 1.47 | 10.33 | 117.18 |  |  |  | 31.33 | 0.15 | 23.34 | 224.3 | 0.26 |  |  |
| birch |  |  | -4.36 | -1.81 | 7.34 | 17.61 |  |  |  | 30.24 | 0.35 | 10.39 | 170.56 | 0.12 |  |  |
| ***AM*** |  |  |  |  |  |  |  |  |  |  |  |  |  |  |  |  |
| beech | 1Nov. | 1.15 |  |  |  |  |  | 1Jan. |  | 29.52 | 745.46 | -0.01 |  |  |  |  |
| oak | 1Nov. | 1.82 |  |  |  |  |  | 1Jan. |  | 73.51 | 445.64 | -0.01 |  |  |  |  |
| birch | 1Nov. | 3.51 |  |  |  |  |  | 1Jan. |  | 33.38 | 946.99 | -0.01 |  |  |  |  |
| ***UM*** |  |  | Ca | Cb | Cc | C^*^ |  |  |  | Fa | Fb |  |  |  |  |  |
| beech | 1-Sep |  | 0.11 | -19.54 | -18.04 | 228.17 | 2May |  |  | -36.95 | 13.42 |  |  |  | 3.18 | -0.01 |
| oak | 1-Sep |  | 0.94 | -27.74 | -21.59 | 30.91 | 30April. |  |  | -16.45 | -19.73 |  |  |  | 54.75 | -0.01 |
| Birch | 1-Sep |  | 1.29 | 17.98 | 7.88 | 22.14 | 13Jan |  |  | -26.46 | -15.12 |  |  |  | 72.5 | -0.01 |
